# Placement of Hemodialysis Catheters with a Technical, Functional, and Anatomical Viewpoint

**DOI:** 10.1155/2012/302826

**Published:** 2012-08-26

**Authors:** Zeki Aydin, Meltem Gursu, Sami Uzun, Serhat Karadag, Emel Tatli, Abdullah Sumnu, Savas Ozturk, Rumeyza Kazancioglu

**Affiliations:** ^1^Department of Nephrology, Haseki Training and Research Hospital, Haseki, Fatih, 34390 Istanbul, Turkey; ^2^Department of Nephrology, Faculty of Medicine, Bezmiale m Vakif University, 34093 Istanbul, Turkey

## Abstract

*Aims*. Vascular access is of prime importance for hemodialysis patients. We aimed to study early complications of hemodialysis catheters placed in different central veins in patients with acute or chronic renal failure with or without ultrasound (US ) guidance. *Material and Methods*. Patients who were admitted to our unit between March 2008 and December 2010 with need for vascular access have been included. 908 patients were examined for their demographic parameters, primary renal disease, and indication for catheterization, type and location of the catheter, implantation technique, and acute complications. *Results*. The mean age of the patients was 60.6 ± 16.0 years. 643 (70.8 %) of the catheters were temporary while 265 (29.2%) were permanent. 684 catheters were inserted to internal jugular veins, 213 to femoral, and 11 to subclavian veins. Arterial puncture occurred in 88 (9.7%) among which 13 had resultant subcutaneous hematoma. No patient had lung trauma and there had been no need for removal of the catheter or a surgical intervention for complications. US guidance in jugular vein and experience of operator decreased arterial puncture rate. *Conclusion*. US-guided replacement of catheter to internal jugular vein would decrease complication rate. Referral to invasive nephrologists may decrease use of subclavian vein. Experience improves complication rates even under US guidance.

## 1. Introduction

Vascular access has prime importance in patients on hemodialysis (HD). Currently, dialysis population consists of older patients who have diabetes mellitus and peripheral obstructive vascular disease. Although autogenous arteriovenous fistulas (AVFs) are the first choice as the permanent vascular access, a period of at least six weeks is recommended to pass after the formation of AVF to be used [1, 2]. Additional time may be necessary for interventional or surgical operation on the AVF to mature it. Prosthetic arteriovenous (AV) grafts can be cannulated within 2-3 weeks from the implantation, although they are not preferred as the primary vascular access. Additionally, AVF may not be appropriate for patients with serious heart failure or chronic respiratory failure [3] or for those with steal syndrome which causes pain and peripheral ischemia [4]. Hence, temporary and permanent cuffed tunneled catheters should be used in these patients and those who need acute HD [5, 6]. Tunneled catheters have decreased the rates of malfunction, infection, and thrombosis significantly when compared to temporary catheters, and should be preferred if the patient would need this access for more than 1 month [7]. Placements of central venous catheters are high risk vascular procedures and require strict aseptic conditions. HD catheters are not only associated with higher risks of long-term complications like central venous stenosis, thrombosis, and infections, but also early interventional complications like arterial puncture, haematoma, and pneumothorax.

The first choice for catheter placement should be the right internal jugular vein and the second choice should be the left internal jugular vein. Ultrasound (US) guidance has been utilized to minimize the risk of arterial puncture [8, 9]. Hence, National Kidney Foundation recommended real-time US to guide insertion of central venous catheters in order to increase the success rate of placement and reduce insertion-related complications and fluoroscopic screening for optimal localization of the catheter tip after insertion of tunneled catheters [2]. Femoral vein may be preferred if the need for central venous access is supposed to be lesser than one week, and in patients who have acute life-threatening conditions like pulmonary edema and serious hyperkalemia [1]. 

This study was aimed to examine the use of catheters as vascular access and the early complications after catheter implantation in a single center.

## 2. Material and Methods

The patients who needed urgent HD or had dysfunction of the current vascular access while on chronic HD program and have been implanted a catheter by the nephrology practitioners in our clinic between 2008 March and 2010 December have been included in this retrospective study. Four nephrology fellows and two nephrologists were practicing at this period of time. These practitioners were accepted as experienced if their practice period is more than six months and the number of catheters that they have placed successfully exceeded twenty. Inexperienced fellows were to use US guidance in all cases of jugular catheter insertion. Experienced fellows were using US when available that means at working hours. In nonworking hours, experienced fellows were able to place catheters with blind technique.

Patients were examined for demographic parameters (age, gender, body mass index), and primary disease causing renal failure, the type of renal failure (acute or chronic), the clinic from which the patient was referred, catheter implantation sites, catheterization technique (blind or US guided), the type of the catheter (permanent or temporary), and complications within the first three days and findings on the control chest X-rays. The rate of arterial puncture in the first six months of the defined period of time was compared with the remaining period for testing the effect of gaining experience.

Before insertion of the catheter, all patients had their complete blood count, prothrombin time, and partial thromboplastin time checked. Fresh frozen plasma was administered if necessary.

### 2.1. Properties of the Catheters

Temporary catheters had double lumens with 11-12 F diameter and were made of polyurethane. Jugular and subclavian catheters were 16 cm in length, and had swan neck shaped ends. Femoral catheters were straight and 20 cm in length to reach inferior vena cava. Permanent tunneled catheters had also two lumens with a diameter of 14-15 F and were made up of silastic/silicon. The length altered (19, 23, or 28 cm) according to the body size of the patient. The dacron cuff of the permanent catheters was about 5 cm away from the exit site which provided a barrier for infections and stability by formation of fibrous tissue around it.

### 2.2. The Choice of the Vein

Right internal jugular vein (anterior or central approach) was the preferred site among patients who would have the first dialysis session if not orthopneic and had no bleeding diathesis. Left jugular vein was chosen if there was thrombus formation or stenosis following prior catheterization. If both jugular veins were thrombotic or stenotic, subclavian vein or femoral vein (orthopneic patients and those with bleeding diathesis) was used. For permanent catheters, right and then left (in case of thrombosis or stenosis of the right one) internal jugular veins were used. The subclavian vein was used only if both veins are obstructed.

### 2.3. Catheterization Technique

Ultrasound guidance was used mostly for insertion of permanent catheters to internal jugular veins. The linear probe of the US was placed to show the internal jugular vein horizontally in the anterior and central approaches. After visualization of the carotid artery in the medial side and the internal jugular vein in the lateral position, the compressibility of the vein and the pulsatility of the artery; catheterization was performed with Seldinger method.

The blind technique which we used for all three veins was based on the palpation of the artery and puncture of the vein at the probable anatomic site followed by insertion of the catheter by the Seldinger method. Our hemodialysis unit serves 24 hours a day when needed. So emergency cases are quite frequent. Ultrasound guidance can be used in cases where the catheter placement is performed at routine working times, namely, between 8 : 00 AM and 5 : 00 PM. But we have no access to this method at hours other than the routine working hours. So ultrasound is used in elective cases while blind technique is used for emergency situations.

All implantations were performed in a section designed for interventional procedures. The skins overlying the desired insertion site were washed, prepared, and draped and then covered with a surgical cloth with the patient in the supine position. After local anesthesia, internal jugular vein was punctured 0.5–1 cm lateral to the carotid artery. Then the guidewire was pushed through the puncture needle followed by removal of the needle. Ultrasound guidance was used if a couple of attempts have been unsuccessful. Finally, the catheter was placed through the guidewire to the internal jugular vein after dilation with the dilators (Seldinger method) [10]. Catheter was aimed to reach atriocaval junction or right atrium. After controlling the patency of the arterial and venous ends of the catheters by drawing blood, both ends were flushed with isotonic saline followed by injection of heparin to the lumens and closure of the lines. The procedure ended with stabilization of the catheter with sutures and bandage after bleeding controls.

Femoral catheterization was performed by blind technique. The leg was slightly abducted and rotated laterally. The vein was punctured with a guide needle about 1 cm medial to the pulsation of femoral artery and 1-2 cm below the inguinal ligament. The puncture needle of the catheter was inserted 3-4 cm below the guide needle as to meet at the vein. Then, the catheter was placed with Seldinger method as described previously. This vein was preferred if the need for central venous access was supposed to be less than a week, and in patients who have acute life threatening conditions like pulmonary edema.

For permanent catheters, the length of the catheter (19, 23, or 28 cm) was decided according to the body size of the patient. The distance between the point of puncture of the internal jugular vein and the end of the tunnel has been measured using guidewire. After the venous puncture and insertion of the guidewire, a small incision of about 5 mm was formed on the pectoral area followed by formation of a tunnel to the site of entrance of guidewire through the skin by a tunneling trochar attached to the end of the catheter and moving the catheter within this tunnel. After dilation of the soft tissues around the guidewire and insertion of the peel-away sheath, the catheter was inserted to the superior vena cava followed by peeling the sheath. After controlling the patency of both lumens and control of bleeding, lumens were saline flushed and filled with heparin and the catheter was fixed by sutures. Patients who had jugular or subclavian catheters were checked by chest X-ray for the position of the catheter and possible complications.

In case of arterial puncture, pressure and cold were applied for at least 10 minutes and the consecutive dialysis session was performed without heparin.

Statistical analysis was carried on by SPSS for Windows version 13.0. Numeric variables were expressed as mean ± standard deviation. Paired Student, *t*-test or Mann Whitney *U* test were used for intergroup comparisons. *P* values less than 0.05 were accepted as statistically significant.

## 3. Results

A total of 908 patients [428 (47.1%) female, 480 (52.9%) male] with a mean age of 60.6 ± 16.0 years were involved in the study. The mean height, weight, and body mass index of the patients were 162 ± 14.9 cm, 69.8 ± 9.1 kg, and 25.6 ± 3.1 kg/m^2^, respectively.

574 (63.2%) patients were referred from nephrology clinics while the remainder (36.7%) from other clinics. Acute renal failure (ARF) was the reason for catheterization in 176 (19.4%) patients and temporary catheters were used for these cases; whereas 732 (80.6%) patients were catheterized due to chronic renal failure (CRF).

The etiologies of renal disease in the CRF group were diabetes mellitus in 263 (36.0%), hypertension in 97 (13.2%), urologic problems (stone, prostatic hyperplasia, and carcinoma, and neurogenic bladder) in 72 (9.8%), chronic glomerulonephritis in 55 (7.5%), chronic pyelonephritis in 39 (5.4%), polycystic kidney disease in 27 (3.7%), renal amyloidosis in 15 (2.1%), and unknown in 164 (22.4%) patients.

437 (48.1%) patients had the first dialysis session after catheterization, whereas 471 patients (51.8%) were already on regular hemodialysis treatment during which they needed a new vascular access due to dysfunction of the prior one. Of them, 198 patients had nonfunctioning AVF and 20 patients had nonfunctioning AV graft due to either thrombosis or stenosis. 212 of the patients were referred to us due to malfunctioning catheters, either permanent or temporary. The cause of the need for catheter placement in 41 patients who were already on hemodialysis treatment was not recorded. Eight cases (0.9%) were on peritoneal dialysis before catheterization. 

Seven patients were given fresh frozen plasma before the procedures due to abnormalities in the coagulation tests.

Temporary and permanent catheters were applied to 643 (70.8%) and 265 (29.2%) patients, respectively. Anatomic locations of the catheters are presented in Table 1.

Blind technique was used in 288 (42,1%) and US guidance in 396 (57.9%) patients during catheterization of the internal jugular vein. US guidance was not used for femoral and subclavian veins. Ratio of US guidance for permanent and temporary jugular catheters was 85.3% and 48.4%, respectively (*P* = 0.001).

Rates of early complications according to the site are presented in Table 2. Arterial puncture occurred only in 48 (7.0%) patients during placement of catheter to the internal jugular vein (11 patients in US guidance and 37 patients in blind technique) and in 39 (18.3%) patients during catheterization of the femoral vein (*P* = 0.001).

When all patients are considered, the rate of arterial puncture in the first six months of the defined period of time was 18.1% which decreased to 6.5% since then (mean 11.3%) (*P* = 0.02). No patient needed removal of the catheter or surgical intervention or lung trauma due to complications of the procedures. 

## 4. Discussion

One of the most important finding of the present study is a relatively low incidence of arterial puncture associated with venous cannulation of the internal jugular vein (7.0%) most of which was under US guidance and a higher incidence of arterial puncture of the femoral vein (18.3%) which was blind. Another important result is that there was also a decrease in complication rate over the course of the study (the rate of arterial puncture in the first six months of the study period was 18.1% which decreased to 6.5% since then). Although, studies similar to ours, including both jugular and femoral vein at the same paper are scarce, the findings of our study are not novel. Several randomized studies report higher incidence of arterial puncture during dialysis catheter placement using anatomical landmarks as opposed to US guided catheter placement in both the internal jugular vein [11–13], as well as in the femoral vein [14–16]. Prabhu et al. [14] demonstrated 18.2% incidence of arterial puncture of the femoral vein compared to 5.5% with US guidance. There have also been review articles in dialysis patients [17] and in patients requiring continuous renal replacement therapies [18]. In a randomized study, US guidance was shown to reduce the risk of arterial puncture significantly (*P* = 0.002) [6]. The rate of arterial puncture was 3.5% when US guidance was used in the present study. Furthermore, a meta-analysis supports use of two dimensional US guidance for catheter placement limited for femoral vein [19].

In our study, arterial cannulation rate of temporary catheters was 10.4%, whereas 7.9% for permanent catheters. Permanent cuffed tunneled catheter placement is a more complicated procedure and this may affect the approach of operator. Moreover, this technique requires relatively elective conditions and more qualified staff. So it may be less frequently preferred in emergency conditions. In our series, blind technique and temporary catheters were preferred mostly in patients at emergency conditions during out-of-work hours, as European Best Practice Guidelines recommended. Hence in our study, most of the temporary catheters were inserted with blind technique (ratio of US guidance for permanent and temporary jugular catheters was 85.3% and 48.4%, resp.). This is why the arterial puncture rate of permanent catheter is lower than temporary catheters.

The experience of the physician is another important factor determining the rate of complications [1]. The procedures are performed by nephrologists and nephrology fellows in our clinic. The decrease in the rate of complication from 18.1% to 6.5% after the first six months (*P* < 0.05) may be related to the increase in the experience of these fellows. This shows the importance of experience in catheter insertion even under US guidance. On the contrary, in their paper Geddes et al. [20] showed no difference between experienced and inexperienced operators when US guidance was used. But they defined operators as “experienced” (>3 years postgraduate and >25 previous cannulation) or “inexperienced” (<3 years postgraduate and less than 3 previous cannulations) which is different than our criterion.

Arterial puncture rate was reported to be higher during catheterization of femoral and subclavian veins in a study carried on in our country [21] which is consistent with our findings (18.3% in femoral catheterization).

Subclavian veins are not routinely used any more due to risk of central venous stenosis [22]. We used subclavian veins only in 11 (1.2%) patients due to thrombosis of the other veins. This low incidence may show favorable approach of an invasive nephrology clinic.

The low complication rate in the present study may be related to the use of internal jugular veins preferentially, experience of the staff and use of US.

## 5. Conclusions

US guided replacement of the catheter to the internal jugular vein would decrease complication rate. Referral to invasive nephrologists may decrease subclavian vein usage for catheter placement. Experience in practice at catheter placement improves complication rates even under US guidance. 

## Figures and Tables

**Table 1:** The anatomic locations of the catheters.

Anatomic locations	Number and percentage of patients	Side	Temporary catheter	Permanent catheter	Total
Internal jugular vein	684 (75.3%)	Right Left	364 62	189 69	553 131
Femoral vein	213 (23.5%)	Right Left	168 45	— —	168 45
Subclavian vein	11 (1.2%)	Right Left	2 2	4 3	6 5

Total	908		643	265	908

**Table 2:** Early complications of central venous catheterization.

Anatomic locations		Arterial puncture (temporary catheter)	Arterial puncture (permanent catheter)	Total	*n* (%)*
Internal jugular vein	Right Left	18 10	11 9	29 19	48 (7.0%)
Femoral vein	Right Left	25 14	— —	25 14	39 (18.3%)
Subclavian vein	Right Left	— —	— 1	— 1	1 (9%)

Total		67 (10.4%)	21 (7.9%)	88	88 (9.7%)

*The percentage within the catheters inserted to the related vein.
